# Effect of silver nanoparticles associated with fluoride on the progression of root dentin caries *in vitro*

**DOI:** 10.1371/journal.pone.0277275

**Published:** 2023-01-20

**Authors:** Flaviana Alves Dias, Cristina M. P. Vidal, Carissa L. Comnick, Xian Jin Xie, Sandrine Bittencourt Berger

**Affiliations:** 1 Department of Restorative Dentistry, University of North Parana, Londrina, Paraná, Brazil; 2 Department of Operative Dentistry, College of Dentistry, The University of Iowa, Iowa City, Iowa, United States of America; 3 Division of Biostatistics and Computational Biology, College of Dentistry, The University of Iowa, Iowa City, Iowa, United States of America; Danube Private University, AUSTRIA

## Abstract

**Objectives:**

To assess the anti-proteolytic effect and potential to inhibit dentin root caries progression of a silver nanoparticle and fluoride solution (CNanoF) in comparison to silver diamine fluoride (SDF).

**Methods:**

48 specimens of root dentin artificial caries lesion were treated with 38% SDF, CNanoF, CNano or F (n = 6 per group). Ph cycling with demineralization and remineralization solutions simulated caries lesion progression. In addition, specimens were incubated with or without bacterial collagenase in the remineralization solution to induce dentin proteolytic degradation. Dentin degradation was assessed by weight loss rate and hydroxyproline (Hyp) release. Changes in cross-sectional microhardness, and lesion permeability and collagen integrity as determined by confocal laser scanning microscopy indicated potential for further demineralization inhibition. The effect of the solutions on the activity of metalloproteinases (MMP) -2 and -9 was also investigated. Statistical analysis consisted of ANOVA, Kruskal-Wallis, and linear mixed models with post-hoc pairwise Tukey, Dunn, and t-tests (α = 0.05).

**Results:**

Treatment with SDF resulted in lower weight loss rate than did other solutions, but all groups showed similar Hyp release (p = 0.183). SDF resulted in greater microhardness at superficial layers of the caries lesions (p<0.05), while there were no differences among CNanoF, CNano, and F. Lesion permeability was similar among all groups after pH cycling (p>0.05), with or without the use of collagenase (p = 0.58). No statistically significant difference was noted among solutions regarding collagen integrity after pH cycling; however, SDF-treated dentin had a significant decrease in collagen integrity when collagenase was used (p = 0.003). Interestingly, only SDF was able to completely inactivate MMP-2 and -9.

**Conclusions:**

CNanoF and SDF both potentially prevent dentin degradation during caries lesion progression *in vitro*; however, SDF was more effective at inhibiting further tissue demineralization.

## Introduction

Root dentin caries has become a greater concern in recent years because of its increasing prevalence among older adults (age 65 and older). The number of natural teeth retained as individuals age has increased, with a 45% decrease in total tooth loss over the last two decades [1]. However, more recent evidence shows that the global burden of untreated caries is shifting from children to older adults, with one of the prevalence peaks at age 70 [2]. As a well-recognized disease, if left untreated, root caries can result in pain, extensive tooth destruction and ultimately tooth loss, which affects the overall health and well-being of older adults. Moreover, management of root caries in older adults poses additional challenges due to several factors that include gingival recession leading to frequent exposure of the cervical root dentin, poor plaque control associated with decreased manual dexterity, increased intake of medications, and systemic diseases that can cause hyposalivation and xerostomia [3, 4].

In root dentin caries, as distinct from coronal caries, demineralization initiates at a higher pH as this substrate has lower mineral content with smaller and more soluble hydroxyapatite crystals [5, 6]. Once demineralization takes place and the dentin is exposed, the extracellular matrix is degraded by host-derived enzymes, such as dentin matrix metalloproteinases (MMPs) [7–9], which will promote tissue breakdown and lesion progression. In addition, the root dentin caries microenvironment and microbiome are unique because root surfaces are exposed and bacterial invasion occurs at earlier stages for this form of caries than in enamel lesions [10].

Silver diamine fluoride (SDF) is an alkaline topical solution that contains fluoride and silver, and it is effective in arresting dental caries [11–13]. This simple, safe, affordable, and non-invasive treatment protects dentin from demineralization by changing the microstructure of minerals and collagen in the demineralized tissue. More specifically, dentin treated with SDF has dense spherical grains on its surface, less collagen degradation, and recovery of mechanical properties, suggesting remineralization of the collagen scaffold at extra- and intra-fibrillar levels [14, 15]. SDF also inhibits MMPs and cysteine cathepsins, mainly when used at high concentrations such as 38% [16, 17]. While 38% SDF inhibits the growth of multispecies cariogenic biofilms including species such as Streptococci, Lactobacilli, and Actinomycetes *in vitro* [18, 19], a clinical study reported no changes in microbial profiles or diversity in SDF-treated and non-treated root dentin lesions [20]. However, the major drawback for SDF is that it darkens the tooth surface, which limits its clinical use to anterior teeth mostly. To address this issue, a combination of SDF with potassium iodide (KI) has been proposed since it removes an amount of residual silver from the tooth surface [21, 22], but discoloration was still shown *in vitro* [12, 21]. Moreover, it has been reported that KI reduces the effectiveness of SDF in arresting or preventing caries [22].

More recently, the use of silver nanoparticles has been proposed to prevent or arrest caries and avoid the black staining or discoloration caused by SDF. When incorporated into a fluoride varnish, silver nanoparticles were as effective as SDF in preventing progression of caries in primary molars in a randomized clinical trial, while causing no dark staining [23]. An *in vitro* study reported dentin remineralization and inhibition of collagen degradation by a solution containing polyethylene glycol-coated silver nanoparticles used in combination with 2.5% sodium fluoride [24]. With the aim of developing an anti-caries solution as an alternative to SDF, a recent study has shown that a silver nanoparticles combined with 2% sodium fluoride experimental solution has antimicrobial effects, is biocompatible, and has the potential to promote enamel remineralization much like SDF, with greater effectiveness than other anti-caries solutions, yet without darkening of the substrate [25]. However, the anti-cariogenic effect of this experimental silver nanoparticles solution in dentin has yet to be evaluated.

Therefore, to confirm the efficacy of silver nanoparticles and fluoride in preventing root caries progression, additional studies are needed. The present study investigated the effects of an experimental silver nanoparticles solution (CNano) applied with or without fluoride (F) on the progression of artificial root caries lesions in comparison to 38% SDF. More specifically, both the potential to limit demineralization and the ability to inhibit or reduce collagen degradation during caries lesion progression were assessed. For that, this study assessed changes in weight loss rate, microhardness at the caries lesion surface and body using cross-sectional microhardness (CSMH), and lesion permeability using confocal laser scanning microscopy (CLSM). The anti-proteolytic potential was determined by changes in weight loss rate and hydroxyproline (Hyp) release during caries lesions progression, and collagen integrity in the lesion body using CLSM. In addition, the potential of inhibiting recombinant MMPs was evaluated by gelatin zymography. Therefore, the null hypotheses tested were that there are no statistically significant differences among the anti-cariogenic solutions used to treat carious dentin regarding: 1) collagen degradation (Hyp and weight loss rate results), 2) collagen integrity and lesion permeability (CLSM results), 3) microhardness (CSMH), and 4) their potential to inhibit MMPs (zymography results).

## Materials and methods

### Root dentin specimen preparation and creation of artificial caries lesion

De-identified human extracted sound third molars were collected following a protocol reviewed and approved by the Local IRB Committee (#2019–08777). Teeth were stored at -20°C until use and for no longer than 3 months. Once sample preparation started, specimens were stored either in deionized (DI) water or in 100% humidity under controlled temperature as required for the different methods. The cervical portion of the root was sectioned parallel to the cementum-enamel junction using two diamond discs under water-cooling in a precision cutting machine (Isomet 1000; Buehler, Lake Bluff, IL, USA) to obtain 4 mm-thick slices. Two root dentin specimens were obtained from each slice (4 mm width × 5 mm length × 5 mm height), which were flattened and polished with silicon carbide papers (Silicon Carbide Grinding Paper; Struers, Cleveland, OH, USA) (grit #600 and #800) under constant water irrigation. Then, the surface of each of the 48 dentin specimens was covered with acid-resistant nail polish (Revlon, New York, NY, USA) leaving a 4 mm × 3 mm exposed area on the dentin surface. To create an artificial caries lesion, dentin specimens were immersed in demineralization solution (2.2 mM CaCl_2_×2H_2_O, 2.2 mM KH_2_PO_4_, 50 mM acetate, pH 4.4) for 96 h at 37°C to induce approximately 70–100 μm-deep lesions as previously described [15]. After demineralization, specimens were rinsed with DI water and half of the caries lesion was covered with the nail polish.

### Treatment with anti-cariogenic solutions

**Fig 1:**
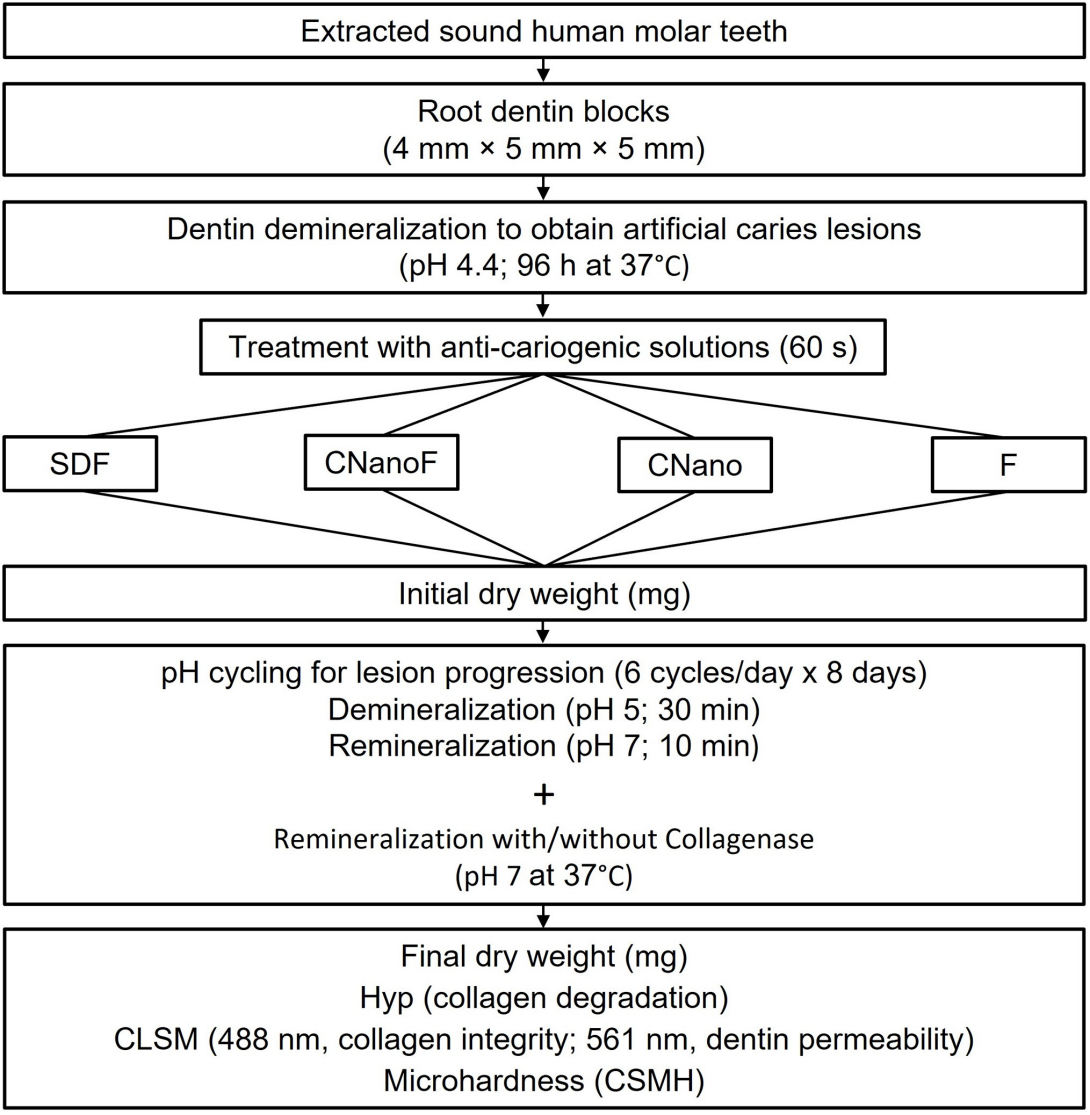
Flowchart depicting the experimental design.

An experimental silver nanoparticles solution (CNano; Biodinamica, Ibipora, Parana, Brazil) was used with or without its fluoride solution component (F; Biodinamica). These solutions are provided separately since earlier studies showed that their combination in a single bottle affects the stability of the nanoparticles [25]. To clarify the isolated effects of the silver nanoparticles and the fluoride, solutions were applied in combination (CNanoF) or separately (CNano and F, respectively). A commercially available 38% SDF solution (Advantage Arrest; Elevate Oral Care, West Palm Beach, FL, USA) was used for comparison. The sample size was selected based on previous studies [15] and a power analysis performed for initial data using G*Power (version 3.1.9.6; Universität Düsseldorf: G*Power (hhu.de); Kiel University; Germany). Results indicated a sample size of 6 (n = 6 per group) to achieve 99% power and to detect a treatment effect size of 1.23 KHN between the group with the highest mean and lowest mean in microhardness using an ANOVA test (α = 0.05). Therefore, after creation of the artificial caries lesions, the specimens were randomly allocated into four experimental groups according to the anti-cariogenic solutions (n = 6): SDF, CNano applied with fluoride (CNanoF), CNano applied without fluoride (CNano), or fluoride solution from CNano (F) (Table 1). Only the surface of the exposed half-lesion was treated with one of the different anti-cariogenic solutions, which were applied for 60 s with a disposable micro-brush. The surfaces were not rinsed or dried with an air syringe after the application. Table 1 shows the composition and pH of all anti-cariogenic solutions used in this study and Fig 1 depicts a flowchart showing the experimental design.

**Table 1:** Composition and pH of the anti-cariogenic solutions used to treat the root dentin caries lesions.

Solutions	Composition	Manufacturer
SDF (Advantage Arrest)	29–32% Silver fluoride (253,900 ppm silver and 44,800 ppm fluoride), 8–10% ammonia, 62.5% water pH 10–10.5	Elevate Oral Care, West Palm Beach, FL, USA
CNano	0.04% colloidal silver nanoparticles with size from 20–100 nm (average size 30nm), ethylene glycol, polyvinylpyrrolidone, water pH 3–5	Biodinamica, Ibipora, Parana, Brazil
F	2% sodium fluoride, water pH 6–8	Biodinamica, Ibipora, Parana, Brazil

### Caries lesion progression (pH cycling)

To assess the protective effect of the solutions against the progression of the artificial caries lesions, specimens were pH cycled at room temperature through six daily cycles of incubation in demineralization and remineralization solutions for 8 days, following a protocol described in previous studies [15, 26]. For that, the nail polish was removed from the entire caries lesion surface and both the non-treated area (NT) (initially covered when the anti-cariogenic solutions were applied) and treated area (T) were exposed to pH cycling. All solutions were made fresh daily prior to use. The demineralization consisted of 30 min immersion in 2.25 mM CaCl_2_×2H_2_O, 1.35 mM KH_2_PO_4_, 130 mM KCl, 50 mM acetate, pH 5. The remineralization was performed by immersion in 20 mM 4-(2-hydroxyethyl)-1-piperazineethanesulfonic acid (HEPES), 2.25 mM CaCl_2_×2H_2_O, 1.35 mM KH_2_PO_4_, 130 mM KCl, pH 7 for 10 min. After the 6 cycles were completed, the dentin specimens were kept in remineralization solution at 37°C overnight with or without bacterial collagenase [15, 27]. Therefore, bacterial collagenase was added to half of the specimens to simulate an enzymatic challenge (100 μg/ml *Clostridium histolyticum*: Sigma-Aldrich, St. Louis, MO, USA). After the overnight incubation, the remineralization solutions containing collagenase were stored at -20°C for further analysis.

### Dentin degradation: Weight loss rate and hydroxyproline assay

The dry weight of each dentin specimen after treatment with the anti-cariogenic solutions (initial weight) and after caries lesion progression (pH cycling) (final weight) was obtained using an analytical balance (XSE205DU; Mettler Toledo, Greifensee, Switzerland) after 24 h of drying in a desiccator. The percentage of weight loss rate was calculated according to the following formula:

$$\text{Weight}\;\text{loss}\;\text{rate}=\frac{\text{Initial}\;\text{weight}-\text{Final}\;\text{weight}}{\text{Initial}\;\text{weight}}\times 100$$

As previously stated, the remineralization solutions containing bacterial collagenase from the overnight incubation were stored and used to quantify the amount of hydroxyproline (Hyp) released from the dentin lesions [28]. For that, a 500 μl aliquot of each solution was lyophilized, re-suspended in 2N NaOH, and hydrolysis was performed at 120°C for 60 min. After, chloramine-T reagent was added and incubated for 25 min at room temperature. The chromophore was developed by incubation with Ehrlich’s reagent for 40 min at 65°C. Absorbance values were measured at 550 nm in a spectrophotometer (SpectraMax M2e; Molecular Devices, Sun Jose, CA, USA) using a standard curve of known concentrations of Hyp. The results were expressed as micrograms of Hyp per milliliter of solution (μg/ml).

### Lesion permeability and collagen integrity using confocal laser scanning microscopy (CLSM)

After the pH cycling was completed, specimens were rinsed with DI water for 2 min and sectioned perpendicularly to the caries lesion surface in two halves using a diamond disc under water cooling (IsoMet 1000; Buehler). Then, the two halves were embedded in epoxy resin (EpoxiCure 2 Resin; Buehler) leaving the cross-sectional lesion area exposed. Specimens were polished with silicon carbide paper (grits #600, #800, and #1200) (Silicon Carbide Grinding Paper; Struers) under constant water irrigation for 3 min in each grit. Diamond suspensions (9, 6, 3, 1, 0.25 μm) (MetaDi diamond suspension polishing; Buehler) were used for polishing for 2 min followed by cleaning for 5 min in an ultrasound water bath in between each solution. Afterwards, the specimens were stained with freshly prepared 0.01% rhodamine B solution (Sigma-Aldrich) and incubated overnight at room temperature. Surfaces were rinsed with DI water and analyzed by CLSM (Zeiss LSM 710; Carl Zeiss, Germany) using an argon laser with two excitation wavelengths: 561 nm (red fluorescence) for dentin micro-permeability evaluation [29], and 488 nm (green fluorescence) for collagen integrity analysis [30]. A differential interference contrast channel was also used to acquire images. From each sample, the regional average fluorescence emission intensities were obtained using software (ZEN 3.0 software, Carl Zeiss) for both T and NT areas at 20, 50, 80, and 110 μm of depth from the lesion surface. For each depth, measurements were made at three different locations and reported as the average of the ratio between T and NT areas.

### Cross-sectional microhardness (CSMH) test

The same samples prepared for the CLSM were also used to evaluate whether changes in cross sectional microhardness indicated protection against demineralization. CSMH test measurements were performed with a Knoop tip (KHN) using 10 g load force for 15 s in microhardness tester (Micromet II Microhardness Tester; Buehler). Measurements were obtained from 20 to 200 μm of depth from the caries lesion surface and three different parallel readings with 100 μm of distance between them for each depth [26]. The three measurements made at three different locations were averaged and reported as the ratio between T and NT areas.

### Gelatin zymography

To further investigate the anti-proteolytic potential of the anti-cariogenic solutions, their inhibitory effect on the activity of MMP-2 and MMP-9 was assessed by gelatin zymography [28]. Recombinant human MMP-2 (62 kDa) and MMP-9 (83 kDa) (Sigma-Aldrich) previously incubated with or without the anti-cariogenic solutions for 10 min at 37°C were diluted in a sample buffer and subjected to electrophoresis under non-reducing conditions in 10% SDS-PAGE containing 1 mg/ml gelatin. After electrophoresis, the gels were washed twice for 30 min in 2% Triton X-100, followed by incubation in activation buffer (50 mM Tris-HCl, 5 mM CaCl_2_, 1 μM ZnCl_2_ pH 7.4) for 48 h. Then, gels were stained with Coomassie Brilliant Blue solution and scanned to obtain images (Azure 500; Azure Biosystems, Dublin, CA, USA).

### Statistical analyses

All statistical analyses were performed using R version 3.6.1 at a 5% significance level. The mean and standard deviation were calculated for all measured parameters. For the Hyp results, a Kruskal-Wallis test with a post-doc Dunn test were used to examine the overall and pairwise differences. ANOVA with Tukey post-hoc tests were performed to calculate differences between groups for weight loss rate. A linear mixed model was used to examine the relationships among depth, collagenase, and anti-cariogenic solution, while accounting for the repeated measurements taken over depths for lesion permeability, collagen fluorescence, and microhardness results. In addition, the ratio of the green and red fluorescence as well as the microhardness results in the T and NT areas of the same samples were compared using t-tests.

## Results

Table 2 presents the weight loss rate and the Hyp release results. In general, the lesions exposed to collagenase had a greater rate of weight loss than those subjected to pH cycling without collagenase (p<0.001). In addition, the ANOVA test showed significant differences among groups (p<0.001) and there were significant pairwise differences between SDF and each of CNano, F, and CNanoF. In all cases, use of SDF resulted in a lower weight loss than each other solution (p<0.05). There were no statistically significant differences in Hyp release among all groups (p = 0.183).

**Table 2:** Mean (standard deviation) of weight loss rate for all groups incubated with and without collagenase and hydroxyproline (Hyp) release of dentin samples subjected to collagenase degradation during pH cycling (n = 6).

Solutions	Weight loss rate (%)		Hyp (μg/ml)
	pH cycling with collagenase	pH cycling without collagenase	
SDF	-0.49 (0.78) ^A, b^	-0.31 (0.42) ^A, a^	1.64 (0.42) ^A^
CNanoF	-1.90 (0.66) ^B, b^	-0.60 (0.72) ^B, a^	1.93 (0.92) ^A^
CNano	-2.14 (0.99) ^B, b^	-1.22 (0.35) ^B, a^	1.92 (1.54) ^A^
F	-2.03 (0.59) ^B, b^	-1.32 (0.86) ^B, a^	1.26 (0.42) ^A^
*p*	<0.001	<0.001	0.183

Different upper-case letters indicate statistically significant difference among anti-cariogenic solutions (p<0.05). Different lower-case letters indicate statistically significant differences between pH cycling with and without collagenase for weight loss rate (p<0.001).

**Fig 2:**
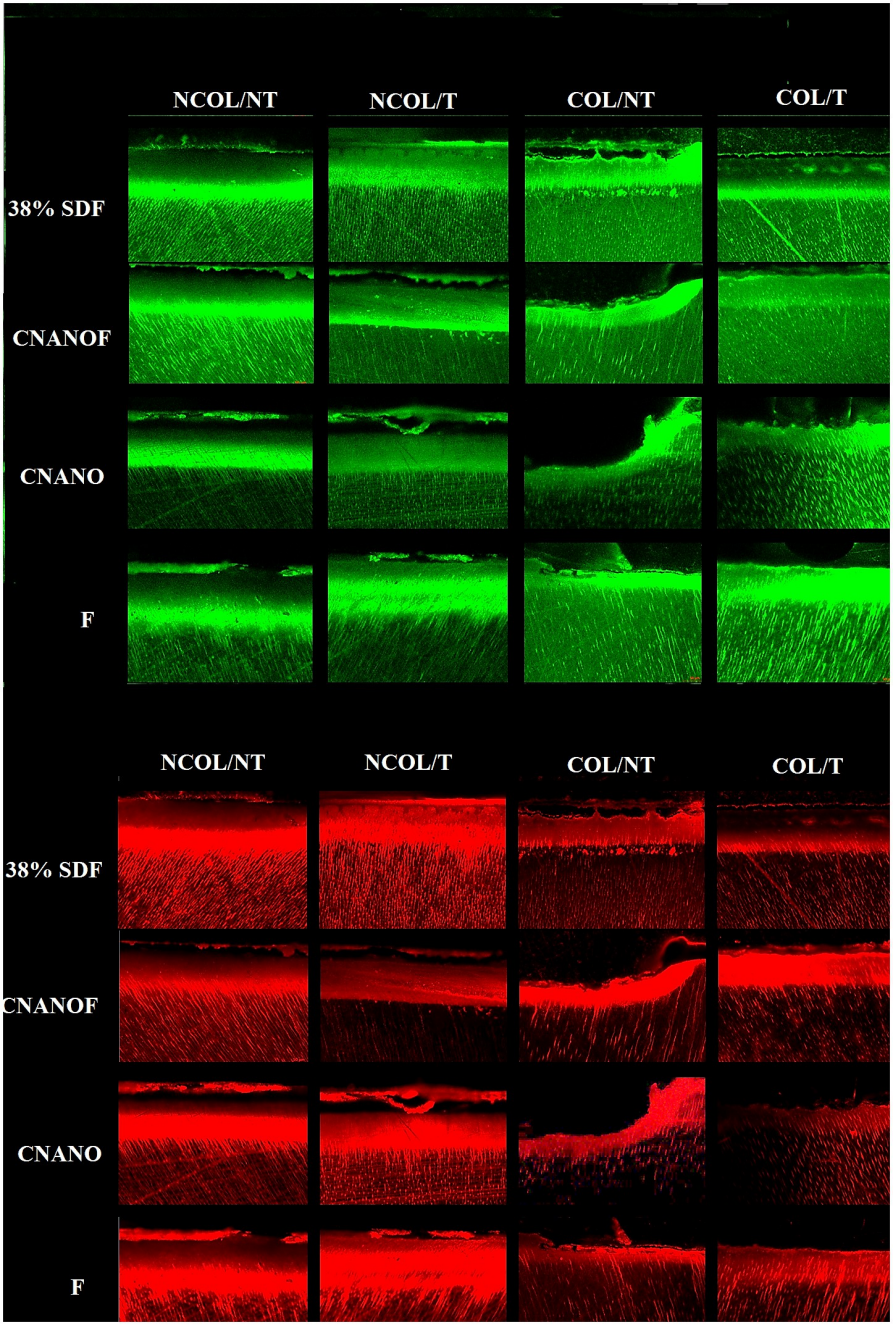
Representative images obtained with confocal microscopy (20x) of cross-sections of dentin caries lesions stained with rhodamine B. Samples were visualized using 561 nm excitation wavelength (red fluorescence) for the quantification of dentin permeability and 488 nm excitation wavelength (green fluorescence) for collagen integrity. T: treated area, NT: non-treated area; COL: lesion subjected to pH cycling with collagenase; NCOL: lesion subjected to pH cycling without collagenase.

Fig 2 shows representative CLSM images of dentin caries lesions subjected to pH cycling with (Col) and without collagenase (NCol). In the NT areas of each lesion, the irregular surface and shallow lesion area indicate that the dentin surface was degraded during pH cycling and caries lesions progressed. When dentin was treated with the different anti-cariogenic solutions, lesion surface was preserved, demonstrating a protective effect against demineralization.

The average emission intensity for the red (lesion micro-permeability) and green (collagen integrity) fluorescence are shown in Tables 3 and 4. Regarding the lesion micro-permeability, the liner mixed model showed no significant pairwise differences between solutions (p>0.05) or between pH cycling with or without collagenase (p = 0.58). For the collagen integrity data, there was significant interaction between solution and addition of collagenase to the pH cycling (p = 0.025). Therefore, the effect of pH cycling with or without collagenase differs by anti-cariogenic solution, as shown in Table 4. After adjusting for the effect of depth, SDF was the only solution that had a significant decrease in collagen integrity when pH cycling was performed with collagenase, while the other groups showed similar results for the two pH cycling conditions (Table 4). The only significant pairwise difference between solutions was between SDF and F solution when collagenase was used. Increased depth was not significantly associated with any change in green (p = 0.15) or red fluorescence (p = 0.270). Regarding the comparison of the ratio between T and NT areas of each sample within solutions for each depth combining the pH cycling conditions, only the CNano and CNanoF at 50 μm showed statistically significant difference for green and red fluorescence, respectively (p = 0.019 and p = 0.030, respectively).

**Table 3:** Mean (standard deviation) of the ratio between T and NT areas of the red fluorescence intensity (561 nm excitation wavelength) used to determine lesion micro-permeability using CLSM (n = 6).

Solutions	pH cycling with collagenase					pH cycling without collagenase				
	20 μm	50 μm	80 μm	110 μm	Average	20 μm	50 μm	80 μm	110 μm	Average
SDF	0.91 (0.37)	0.79 (0.48)	1.43 (0.76)	0.80 (1.28)	0.98 ^A, a^ (0.80)	1.56 (1.82)	1.47 (0.85)	1.03 (0.24)	0.88 (0.44)	1.23 ^A, a^ (1.01)
CNanoF	1.15 (0.76)	1.61 (0.72)	1.72 (1.14)	1.25 (0.32)	1.43 ^A, a^ (0.78)	1.15 (0.66)	1.28 (0.51)	1.02 (0.28)	1.44 (1.46)	1.23 ^A, a^ (0.81)
CNano	1.22 (0.59)	1.57 (0.59)	1.14 (1.18)	0.69 (0.59)	1.15 ^A, a^ (0.80)	1.03 (0.14)	1.03 (0.16)	0.90 (0.16)	0.82 (0.22)	0.95 ^A, a^ (0.19)
F	0.77 (0.20)	1.71 (0.73)	1.26 (0.72)	0.87 (0.55)	1.15 ^A, a^ (0.67)	1.21 (0.44)	0.93 (0.30)	1.01 (0.21)	1.19 (0.19)	1.09 ^A, a^ (0.31)

Same lower-case letters indicate no statistically significant difference in the mean between pH cycling without and with collagenase after adjusting for the effects of depth for each solution (p>0.05). Same upper-case letters indicate no statistically significant difference in the pairwise comparison between solutions in each condition for pH cycling (p>0.05).

**Table 4:** Mean (standard deviation) of the ratio between T and NT areas of the green fluorescence intensity (488 nm excitation wavelength) used to determine collagen integrity using CLSM (n = 6).

Solutions	pH cycling with collagenase					pH cycling without collagenase				
	20 μm	50 μm	80 μm	110 μm	Average	20 μm	50 μm	80 μm	110 μm	Average
SDF	0.54 (0.13)	0.47 (0.14)	1.29 (0.79)	0.90 (0.42)	0.80 ^A, b^ (0.54)	1.18 (1.35)	1.33 (0.56)	1.27 (0.34)	1.24 (0.64)	1.26 ^A, a^ (0.76)
CNanoF	0.94 (0.59)	1.19 (0.42)	1.18 (0.58)	1.11 (0.25)	1.10 ^A, B, a^ (0.46)	0.94 (0.35)	0.88 (0.19)	1.18 (0.18)	1.10 (0.75)	1.03 ^A, a^ (0.42)
CNano	0.94 (0.24)	1.42 (0.54)	0.94 (0.47)	1.06 (0.48)	1.09 ^A, B, a^ (0.46)	1.13 (0.23)	1.26 (0.33)	1.05 (0.11)	1.12 (0.31)	1.14 ^A, a^ (0.25)
F	0.67 (0.11)	1.81 (0.79)	1.35 (0.38)	1.27 (0.53)	1.27 ^B, a^ (0.63)	1.39 (0.34)	0.91 (0.14)	1.01 (0.38)	1.19 (0.12)	1.13 ^A, a^ (0.32)

Different lower-case letters indicate statistically significant difference in the mean between pH cycling with and without collagenase after adjusting for the effects of depth within each solution (p<0.05). Different upper-case letters indicate statistically significant difference in the pairwise comparison between solutions in each condition for pH cycling (p<0.05).

The microhardness results are shown in Tables 5 and 6. Since there was a consistent increase in microhardness in all groups at 140, 170, and 200 μm, with differences only for the more superficial layers of the lesions, only the results from 20 to 110 μm are reported. The linear mixed model showed significant interactions between depth and each of the solution and pH cycling conditions. In general, microhardness at more superficial depths (20, 50, and 80 μm) was reduced for the samples subjected to pH cycling with collagenase in comparison to without collagenase (p<0.05), with no statistically significant difference at 110 μm (p = 0.976) (Table 5). The pairwise comparison between the different solutions for each depth showed a statistically significant increase in microhardness for SDF than for the other groups at 20 and 50 μm (Table 6). Considering T and NT ratios in the same sample and the results of pH cycling conditions, SDF and CNanoF showed statistically significant difference at all depths, except for 50 μm for CNanoF (p<0.05) (Table 6).

**Table 5:** Mean (standard deviation) of the ratio between T and NT areas of cross-sectional microhardness results for the different anti-cariogenic solutions (n = 6).

Solutions	Depth							
	20 μm		50 μm		80 μm		110 μm	
	Col	NCol	Col	NCol	Col	NCol	Col	NCol
SDF	1.11 (0.12)	1.35 (0.10)	1.12 (0.14)	1.26 (0.19)	1.08 (0.16)	1.34 (0.12)	1.19 (0.08)	1.14 (0.16)
CNanoF	1.09 (0.06)	1.10 (0.12)	1.01 (0.15)	1.16 (0.15)	1.08 (0.05)	1.20 (0.14)	1.11 (0.06)	1.09 (0.16)
CNano	1.01 (0.19)	1.09 (0.14)	0.92 (0.10)	1.04 (0.12)	0.99 (0.08)	1.03 (0.09)	1.04 (0.10)	1.01 (0.07)
F	1.00 (0.11)	1.35 (0.19)	0.87 (0.08)	1.04 (0.23)	0.95 (0.04)	0.94 (0.10)	0.98 (0.05)	0.96 (0.05)
**Average for all solutions within each depth**	1.05 ^B^ (0.13)	1.22 ^A^ (0.19)	0.98 ^B^ (0.15)	1.12 ^A^ (0.19)	1.02 ^B^ (0.10)	1.13 ^A^ (0.19)	1.08 ^A^ (0.10)	1.05 ^A^ (0.13)

Different letters represent statistically significant difference between pH cycling with and without collagenase in the average for all depths (p<0.05). COL: lesions subjected to pH cycling with collagenase; NCOL: lesions subjected to pH cycling without collagenase.

**Table 6:** Mean (standard deviation) of the ratio between T and NT areas of the cross-sectional microhardness for the different anti-cariogenic solutions combining the results for pH cycling with and without collagenase.

Solutions	20 μm	50 μm	80 μm	110 μm
SDF	1.23 ^A,^* (0.17)	1.19 ^A,^* (0.18)	1.21 ^A,^* (0.19)	1.16 ^A,^* (0.12)
CNanoF	1.10 ^B,^* (0.09)	1.09 ^B,^ (0.16)	1.14 ^A,^* (0.12)	1.10 ^A, B,^* (0.12)
CNano	1.05 ^B^ (0.16)	0.98 ^B^ (0.12)	1.01 ^C^ (0.08)	1.03 ^B, C^ (0.08)
F	1.18 ^B,^ * (0.23)	0.95 ^B^ (0.19)	0.95 ^C,^ * (0.08)	0.97 ^C^ (0.05)

Different letters represent statistically significant difference among anti-cariogenic solutions within each depth (p<0.05). Asterisks indicate statistically significant difference in the ratio of microhardness between T and NT areas (p<0.05).

**Fig 3:**
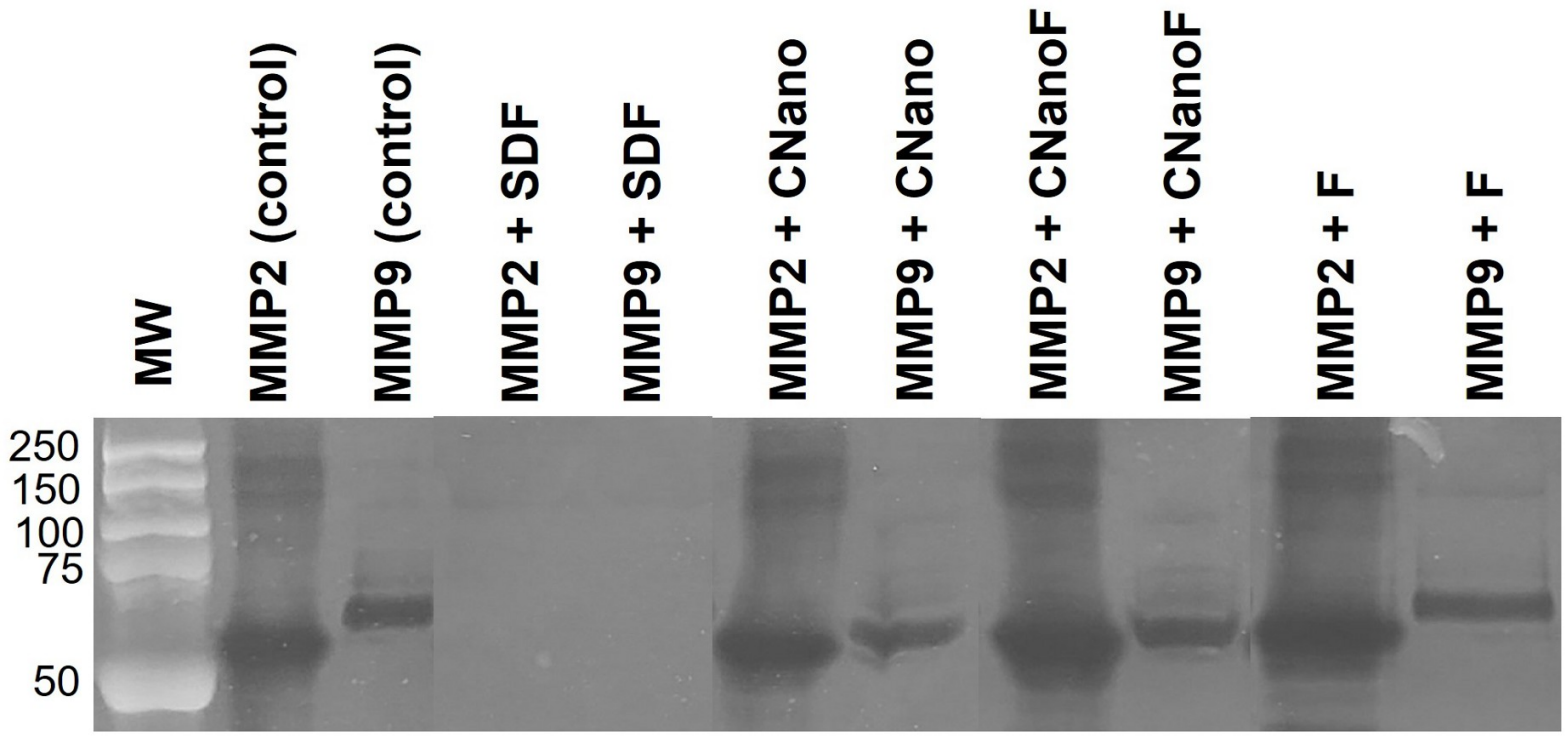
Gelatin zymography of MMP-2 and MMP-9 controls and incubated with the anti-cariogenic solutions. MW: molecular weight standard (from 250 to 50 kDa).

Fig 3 shows a gelatin zymography gel of recombinant human MMP-2 and MMP-9 incubated with or without the anti-cariogenic solutions. Only SDF was able to completely inactivate these enzymes as no bands are observed at their corresponding molecular weights. All other solutions showed similar intensity of gelatinolytic activity for both enzymes tested.

## Discussion

The present study investigated the effects of an experimental silver nanoparticle solution used in combination with fluoride (CNanoF) on the progression of artificial caries lesions in root dentin in comparison to the widely used 38% SDF. According to our results, similar collagen degradation was observed for CNanoF and SDF (Hyp release data), although SDF showed lower weight loss rate than the other three solutions, so we reject the first null hypothesis. Treating the root dentin with SDF or CNanoF resulted in similar collagen integrity and lesion permeability, but SDF showed greater potential to protect the lesion surface from demineralization during simulated caries lesion progression; therefore, the second null hypothesis is rejected, but the third null hypothesis is accepted. Finally, the fourth null hypothesis is rejected since SDF was the only solution to completely inactivate the recombinant human MMPs.

Dentin degradation was assessed by two methods used to estimate carious tissue breakdown during the lesion progression protocol (pH cycling with/without collagenase). The weight loss results reflect changes in both organic and inorganic dentin components during pH cycling, while the quantification of Hyp release is a more accurate method to measure collagen degradation. Contrary to our findings, dentin treatment with polyethylene glycol-coated silver nanoparticles resulted in less Hyp release than SDF in a previous study [24]. However, it is important to consider that the dissimilar findings between our results and the literature might be related to the different experimental silver-nanoparticle preparations, the concentration of silver and fluoride in these solutions, and the comparisons between silver nanoparticles solutions with 12% [24] and 38% SDF (tested in the present study). Possibly, the greatest protection against dentin degradation promoted by SDF during pH cycling is related to its high concentration of silver and fluoride, which is discussed in detail as follows. Interestingly, while all solutions presented similar potential to protect collagen from degradation, dentin treatment with SDF resulted in lower weight loss rate than did other solutions. It may be that SDF affected both the organic and inorganic components of the dentin, and its lower weight loss could be a result of a protective effect against further demineralization, which is supported by the microhardness results.

Together with lesion permeability, collagen integrity was also assessed by CLSM. As shown in Table 4, the greatest reduction in collagen integrity by pH cycling with collagenase was observed for SDF, especially at the more superficial layers of the lesions (Fig 2). Since a prior *in vitro* study showed less collagen degradation in lesions treated with SDF when using a biofilm challenge to simulate lesion progression [31], our findings should be interpreted with caution. Possibly, the bacterial collagenase used in this study promoted a harsh condition and intense dentin degradation, which might have resulted in the poor performance of SDF regarding collagen integrity and similar degradation for all groups (Hyp results). In addition, while pH cycling combined with bacterial collagenase has been widely used to simulate caries lesion progression, it presents limitations as it does not mimic the complexity of caries disease, which might have affected the anti-cariogenic effect of SDF as reported previously [19, 31].

However, SDF showed similar results for all solutions for collagen integrity when pH cycling was performed without the proteolytic challenge. This finding indicates that CNanoF and CNano have also exerted some protection against lesion progression. Interestingly, while treating the dentin with SDF or CNano (with or without F) resulted in similar lesion permeability (Table 3), SDF had greater potential to inhibit surface demineralization than did CNanoF, as demonstrated by the microhardness results (Tables 5 and 6). High mineral levels on SDF-treated surfaces have been widely reported in the literature [14, 15, 18], with some studies suggesting a remineralization effect [31, 32]. Still, it should be noted that greater microhardness numbers might not reflect remineralization, but they do indicate protection against mineral removal during pH cycling. Different mechanisms have been proposed to characterize the effects of SDF on dentin mineral content and include absorption of calcium, production of calcium fluoride and metallic silver, increase in phosphorus content, and greater uptake of fluoride [33]. The precipitation of silver chloride in SDF-treated dentin may also act as a protective layer to reduce mineral loss [31]. In addition, SDF might provide an alkaline environment that facilitates the formation of covalent bonds between the phosphate groups on proteins, thus providing a favorable setting for crystallites to grow [34]. Possibly, greater mineral levels in SDF-treated surfaces may also maintain collagen integrity, which would explain the lower Hyp release as mentioned previously. Curiously, as already mentioned, the proteolytic challenge with collagenase in the pH cycling affected the performance of SDF only. SDF’s reduced microhardness numbers resulting from bacterial collagenase use may suggest that the absence of a preserved collagen did not allow mineral crystal growth to occur, a requirement for proper remineralization [35]. This is supported by the results of collagen integrity measured by the changes in green fluorescence (Table 4). Regarding the changes in mineral levels by silver nanoparticles, controversial findings are reported in the literature [13, 36]. The presence of granuliform structures on dentin treated with silver nanoparticle solution containing 2.5% sodium fluoride suggests possible remineralization [24]. However, when compared to 38% SDF, the silver nanoparticles and fluoride solution resulted in less microhardness [37], which is consistent with our results. In addition, as observed in this study, silver nanoparticles potentially improve collagen chemical structure and stability, but do not protect lesion surface from demineralization [38].

Lastly, SDF was the only solution tested in this study to completely inactivate recombinant MMP-2 and -9 (Fig 3), which has been shown previously [17]. Even though this inhibitory potential did not result in protection of collagen integrity, it might be of clinical relevance. Many studies proposing the use of silver nanoparticles to manage initial caries emphasize their antibacterial properties [13, 37]. Possibly, the most positive effect of silver nanoparticles in arresting lesion progression in dentin relies on its ability to inhibit bacterial activity. In clinical studies, silver nanoparticles solutions have effectively managed lesions [13, 23]. In addition, the high concentration of silver and fluoride in 38% SDF seems to be related to inhibition of MMP. Our findings here and prior studies using lower concentrations suggest that SDF’s inhibition of MMPs is concentration-dependent, which is greatly reduced when the concentration is 30% and 12% rather than 38% SDF [17]. While 5,000 ppm sodium fluoride inhibits salivary and purified MMPs [39], minimum or no inhibition is observed in the gelatin zymography by the F solution in this study.

In fact, differences in silver and fluoride concentration should be considered for the interpretation of all the results from this study. For example, 38% SDF showed more protection against surface demineralization than did 12% SDF [11]. Although 38% SDF contains around 50,000 ppm of fluoride and 260,000 ppm of silver, CNanoF has 1% or 10,000 ppm of fluoride in combination with 1,600 ppm of silver nanoparticles, which might not be enough to express its excellent anti-cariogenic potential. Higher concentration of silver nanoparticles and fluoride should be tested in future studies as different experimental formulations might show greater protection against caries progression.

In summary, the development of anti-cariogenic solutions that have the same potential as SDF to prevent/arrest root caries without staining teeth will open new venues in dental research to improve oral health in a cost-effective and non-invasive way. Although color change was not assessed in this study, dentin treated with CNanoF has significantly less staining as already described [25] than did SDF, which resulted in dark and black surfaces. This study is the first to characterize changes in carious dentin after treatment with CNanoF, which is a necessary step in the development of silver nanoparticle-based solutions to manage root caries. However, the use artificially created caries lesion and pH cycling with collagenase limits our findings. Other methods to simulate the complexity of the oral environment for lesion progression should be explored to clarify the potential anti-cariogenic effect of silver nanoparticles used with fluoride. In addition, future studies should investigate the inhibitory mechanism of endogenous enzymes in a silver nanoparticles solution by studying the potential inhibition of proteases from dentin instead of using recombinant enzymes only.

## Conclusion

Taken together, the results from this study indicate that both CNanoF and SDF have the potential to prevent dentin from degrading during caries lesion progression; however, SDF offered the greatest protection from further demineralization. Therefore, considering the complexity of caries progression *in vivo*, SDF has better anti-cariogenic effects than does CNanoF.

## Supporting information

S1 Raw images (PDF)

S1 Data (XLS)
