# Supplementary material for: Effect of silver nanoparticles associated with fluoride on the progression of root dentin caries in vitro
Source: PLoS One. 2023 Jan 20;18(1):e0277275. doi: 10.1371/journal.pone.0277275 (PMC9858332; doi:10.1371/journal.pone.0277275)
Supplement: S1 Raw images — (PDF) [file pone.0277275.s001.pdf]

Supporting Information (original images for gel)

The image below shows the raw image of the gelatin zymography gel presented in Figure 3. Figure 3 depicts bands from two different gels ran under the same conditions as described in the materials and methods. Numbers on top of the lanes represent the loading order and lanes marked with an “X” represent samples that were not included in the final figure.

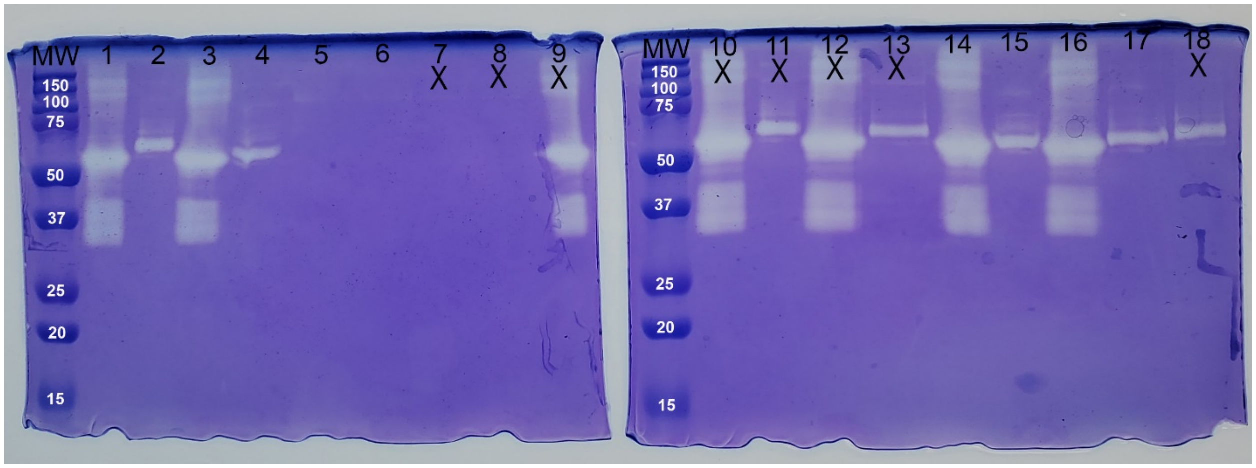

Raw image of gelatin zymography gels ran with recombinant human MMP-2 and -9 incubated with the different anti-caries solutions. MW: molecular weight (Precision Plus Protein Dual Color Standard; Bio-Rad, Hercules, CA, USA); 1: MMP-2 recombinant (62 kDa) (control); 2: MMP-9 recombinant (82 kDa) (control); 3: MMP-2 + CNano; 4: MMP-9 + CNano; 5: MMP-2 + SDF; 6: MMP-9 + SDF; 7-9: additional samples not included in the final image; 10: MMP-2 recombinant (62 kDa) (control) not included in the final image; 11: MMP-9 recombinant (82 kDa) (control) not included in the final image; 12: MMP-2 + CNano not included in the final image; 13: MMP-9 + CNano not included in the final image; 14: MMP-2 + CNanoF; 15: MMP-9 + CNanoF; 16-18: additional samples not included in the final image.

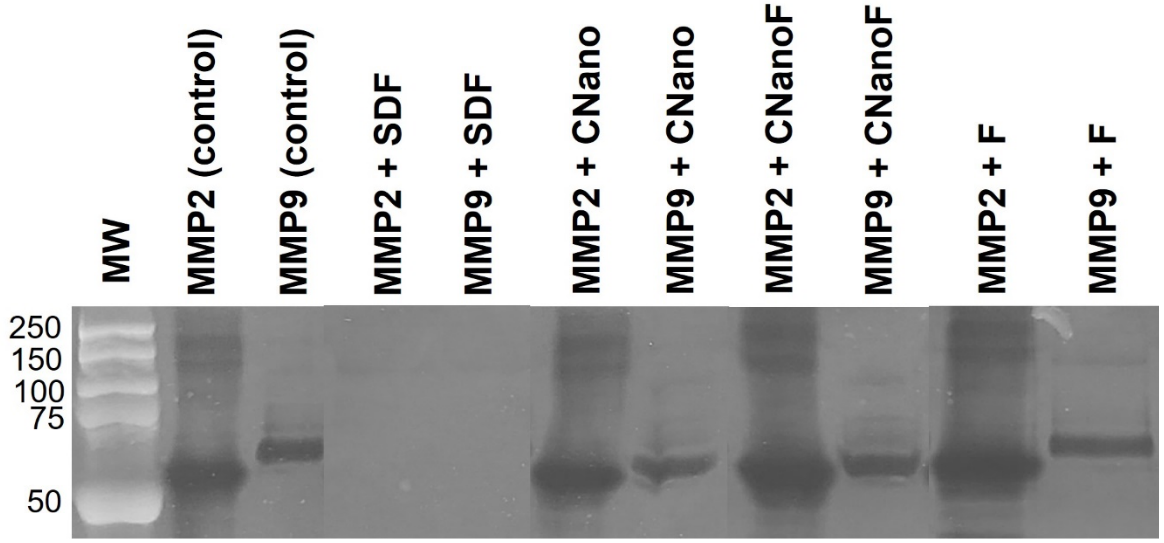

Figure 3. Gelatin zymography of MMP-2 and MMP-9 controls and incubated with the anti-cariogenic solutions. MW: molecular weight standard (from 250 to 50 kDa).
